# Transactions

**Published:** 1879-05

**Authors:** 


### TRANSACTIONS.

The transactions of the eighteenth annual meeting of the American Dental Association is at hand. It presents the doings of that body in a good and an attractive form. The matter throughout is valuable and interesting.

The volume should be in the hands of every dentist. It can be obtained by addressing Dr. Geo. H. Cushing, chairman of the publication committee, Chicago, Ill.

